# Artificial intelligence-assisted radiotherapy for pelvic and abdominal malignancies: assessing feasibility in the context of Africa-specific risks

**DOI:** 10.3389/fonc.2026.1686296

**Published:** 2026-05-26

**Authors:** Emmanuel Fiagbedzi, George F. Acquah, Alhassan M. Baidoo, Linda Osei- Poku, Mark Pokoo-Aikins, Theresa Dery, Annette Agyabeng, Shirazu Issahaku, Theophilus A. Sackey, Mary Adu-Poku, Edem K. Sosu, Samuel Tagoe, Eric C. K. Addison, Francis Hasford, Magdalena Stoeva

**Affiliations:** 1 Department of Medical Imaging Technology and Sonography, University of Cape Coast, Cape Coast, Ghana; 2 RPS Oncology, Minneapolis, MN, United States; 3 Sweden Ghana Medical Centre (SGMC), Accra, Ghana; 4 Ghana Atomic Energy Commission, Accra, Ghana; 5 National Centre for Radiotherapy Oncology and Nuclear Medicine, Korle Bu Teaching Hospital, Accra, Ghana; 6 Oncology Directorate, Komfo Anokye Teaching Hospital, Kumasi, Ghana; 7 Medical University, Plovdiv, Bulgaria

**Keywords:** abdominal malignancies, Africa, artificial intelligence, cancer care, pelvic malignancies, radiotherapy

## Abstract

Cancer care in Africa remains severely limited, with pelvic and abdominal malignancies contributing substantially to the disease burden. Radiotherapy is essential but constrained by infrastructure deficits, workforce shortages, and systemic inequities. Artificial intelligence (AI) may help strengthen radiotherapy through automation and improved workflow efficiency. This narrative review summarises current evidence on AI assisted radiotherapy for pelvic and abdominal cancers in Africa, highlighting feasibility, and regional specific implementation risks. The review shows that AI tools for auto-contouring, treatment planning support, quality assurance, and workflow optimisation can improve efficiency and ease workload when implemented within appropriate clinical and governance frameworks. Their clinical impact in African radiotherapy, however, is constrained by limited digital infrastructure, workforce shortages, weak data governance, regulatory gaps, and poor model generalisability. Additional risks including data bias from non-African training datasets, and fragile IT systems underscore the need for cautious deployment. A feasibility-first, phased adoption strategy centred on hybrid AI–human workflows, regional model validation, workforce upskilling, and policy-led governance offers a safe and practical route for integrating AI into African radiotherapy. When integrated within resilient systems and guided by risk-aware strategies, AI has the potential to act as a capacity multiplier rather than a substitute, offering a more equitable access to high quality radiotherapy across Africa.

## 1 Background

Pelvic and abdominal malignancies account for a substantial proportion of the cancer burden in Africa and remain major contributors to cancer-related morbidity and mortality. Cancers of the cervix, prostate, colorectum, liver, ovary, bladder, stomach, and other abdominopelvic organs are frequently diagnosed at advanced stages, resulting in poor survival outcomes and significant socio-economic impact (1, 2). Data from global cancer surveillance initiatives, including GLOBOCAN, indicate disproportionately high mortality-to-incidence ratios in African countries compared with high-income regions (2, 3). Cervical cancer remains the dominant pelvic malignancy in sub-Saharan Africa, while prostate and colorectal cancers are increasing in incidence, particularly in urban populations (4, 5). In North Africa, including Egypt, liver and colorectal cancers represent a substantial disease burden (3, 6, 7). This disproportionate burden reflects persistent gaps in prevention, early detection, and access to effective treatment, in contrast to high-income regions where organised screening, vaccination programs, and timely oncologic care have significantly reduced mortality (8). Cultural barriers, inadequate diagnostic infrastructure, and poor referral pathways further compound these disparities, leading to late presentation in many African settings (9–11).

Radiotherapy is a key component of cancer management for pelvic and abdominal malignancies, with more than half of all cancer patients requiring radiotherapy at some point during their treatment course (12). In Africa, however, access to radiotherapy services remains severely constrained by limited and uneven distribution of infrastructure, as well as critical shortages of trained radiation oncologists, medical physicists, and radiation therapists (13, 14). Outdated equipment, frequent machine downtime, and limited quality assurance capacity further compromise treatment delivery. These systemic constraints leads to prolong waiting times, treatment interruptions, and suboptimal clinical outcomes, particularly for patients in rural and underserved regions (15).

Against this backdrop, artificial intelligence (AI) is increasingly recognised as a potential enabler for strengthening radiotherapy services through automation, workflow optimisation, and enhanced clinical decision-making. AI-assisted applications in image segmentation, treatment planning, quality assurance, and adaptive radiotherapy have demonstrated efficiency gains and improved consistency in well-resourced settings (16, 17). Conceptually, these tools have the potential to ease workforce pressures, shorten planning times, and support more standardised care in resource-limited African radiotherapy centres. However, translating these AI-driven advances into meaningful radiotherapy capacity improvements and outcomes in Africa is neither straightforward nor guaranteed. Effective AI implementation depends on robust digital infrastructure, reliable data governance frameworks, adequate technical expertise, and sustained institutional support (18). For pelvic and abdominal radiotherapy, where organ motion, anatomical variability, and varying dose constraints are highly variable, the performance and safety of AI systems require careful assessment and adaptation.

This narrative review examines the emerging role of artificial intelligence (AI) in radiotherapy for pelvic and abdominal malignancies in Africa, highlighting the current limited efforts such as automated contouring tools and AI-assisted treatment planning. The focus is on practical feasibility, addressing African-specific risks, and exploring mitigation strategies. By drawing on available evidence and regional experiences, the review aims to guide clinicians, policymakers, and stakeholders in responsibly integrating AI-enabled radiotherapy into cancer care systems across the continent.

## 2 Methods: literature identification and scope

This narrative review was conducted as an examination of complex, heterogeneous evidence across technical development, clinical implementation, health-systems feasibility, and policy considerations related to AI-assisted radiotherapy. A narrative review methodology was chosen because the literature spans diverse study designs (technical validation studies, feasibility reports, implementation case studies, and policy analyses) that are not suitable for meta-analysis or systematic synthesis.

### 2.1 Literature search strategy

A structured search was performed across PubMed/MEDLINE, Scopus, and Web of Science to identify peer-reviewed studies on addressing AI applications in radiotherapy for pelvic and abdominal malignancies. The search strategy combined terms related to:

- artificial intelligence (“artificial intelligence”, “machine learning”, “deep learning”);
- radiotherapy (“radiotherapy”, “radiation oncology”, “treatment planning”, “adaptive radiotherapy”, “quality assurance”);
- workflow applications (“auto-contouring”, “auto-segmentation”, “dose prediction”, “plan optimisation”, “image registration”, “patient-specific QA”); and
- geographic or contextual focus (“Africa”, “sub-Saharan Africa”, “low- and middle-income countries”).

The search covered publications from January 2010 to August 2025 to capture contemporary AI techniques while retaining foundational literature on radiotherapy access and workforce challenges in Africa.

### 2.2 Eligibility criteria

Studies were eligible if they met the following criteria:

✔ addressed AI-enabled applications within radiotherapy workflows (contouring, planning, adaptive radiotherapy, quality assurance, workflow optimisation);
✔ focused on pelvic or/and abdominal malignancies, or radiotherapy systems directly applicable to abdominopelvic treatment;
✔ reported data, validation, feasibility, or implementation considerations relevant to African settings or explicitly discussed transferability to low and middle-income countries.
✔ Peer-reviewed original research articles, reviews, and guidance papers were included. Non-peer-reviewed studies and publications unrelated to radiotherapy were excluded.

### 2.3 Study selection and synthesis

Articles were selected through title and abstract-level screening for relevance, followed by full-text review. Eligible studies were required to report on AI application in radiotherapy, with reported information on performance or validation approaches, infrastructure and data requirements, workforce implications, and feasibility constraints specific to African radiotherapy settings. Given the heterogeneity of study design and outcomes, eligible findings were synthesised thematically rather than quantitatively, in line with best practice guideline for narrative reviews addressing complex healthcare interventions.

### 2.4 Scope and analytic focus

The review prioritises ongoing AI developments provided they offer insights into emerging trends relevant to radiotherapy in Africa. Its scope also included implementation feasibility and risk-aware integration rather than technological capability alone. Evidence from high-income settings was included solely when it offered potentially transferable insights, but interpretation was explicitly framed around African contextual realities such as infrastructure limitations, workforce capacity, data governance, and sustainability concerns (16, 19).

## 3 Results synthesis and discussion

The structured literature search identified a heterogeneous body of peer-reviewed publications addressing AI applications within radiotherapy workflows relevant to pelvic and abdominal malignancies. Following screening and full-text assessment in accordance with predefined eligibility criteria, the included literature comprised technical validation studies, feasibility and implementation reports, multi-institutional reviews, and governance analyses. The majority of AI technical validation studies originated from high-income regions, whereas African-specific literature primarily consisted of feasibility analyses, readiness assessments, and pilot implementation reports. Given the narrative methodology, no quantitative pooling was performed. Instead, findings were synthesised thematically across three principal domains: (1) AI applications relevant to pelvic and abdominal radiotherapy workflow; (2) feasibility determinants for AI implementation and (3) African-specific risks and mitigation strategies; as well as lessons from Africa.

### 3.1 AI application relevant to pelvic and abdominal radiotherapy workflow

The synthesis identified four workflow areas of AI application relevant to pelvic and abdominal radiotherapy: auto-contouring, treatment planning, adaptive radiotherapy (ART), and quality assurance (QA) (16, 20).

#### 3.1.1 Auto-contouring (segmentation of targets and organs-at-risk)

Auto-contouring is the most common AI application identified across the literature. Deep learning-based segmentation models have demonstrated reductions in contouring time and improvements in inter-observer consistency for pelvic targets and organs-at-risk (OARs) (21). Pelvic malignancies, particularly cervical and prostate cancers, were the most frequently evaluated sites. In several studies, automated segmentation achieved geometric agreement within clinically acceptable thresholds; however, performance varied across datasets and institutions (22–26). Consistent across reports was the recommendation that AI-generated contours undergo clinician review prior to clinical approval. The evidence supports implementation within hybrid AI–human workflows rather than fully autonomous deployment (27–29). Web-based and LMIC-oriented platforms such as the Radiation Planning Assistant (RPA) have been developed to automate contouring and planning steps for resource-limited clinics, supporting standardised processes where specialist time is scarce (30). Gynaecological brachytherapy, a form of radiotherapy in which contouring and planning are highly expertise-dependent, has seen the implementation of self-configuring nnU-Net approaches for automatic delineation of targets and OARs in cervical cancer HDR workflows, explicitly highlighting applicability in low/middle-income settings (31).

#### 3.1.2 Treatment planning (plan generation, dose prediction, and optimisation)

Evidence from cervical cancer radiotherapy demonstrates that automated planning can generate clinically acceptable plans, supporting the feasibility of planning automation in settings with limited specialist capacity (32). AI-based planning aims to reduce planning time while maintaining clinical acceptability and consistency. Broader reviews also describe AI-enabled dose prediction and optimisation as an advancing field, increasingly integrated with adaptive workflows and decision support (20, 33).

#### 3.1.3 Adaptive radiotherapy

Within African radiotherapy settings, ART was infrequently reported outside pilot initiatives, reflecting substantial infrastructure and training requirements. ART relies heavily on AI-driven deformable image registration and motion modelling. Pelvic and abdominal sites present specific challenges due to inter-fraction and intra-fraction anatomical variability, including bladder filling, rectal distension, and respiratory motion (34, 35). AI-enabled ART has demonstrated feasibility in digitally mature environments. However, implementation requires advanced imaging capabilities, computational infrastructure, real-time workflow integration, and robust quality assurance processes (34).

#### 3.1.4 Quality assurance

QA is another major target for AI because it consumes substantial physics time and generates high-dimensional data (plan parameters, logfiles, dose distributions, gamma maps). Machine learning models have been used to predict patient-specific QA outcomes and flag plans likely to fail tolerance thresholds before measurement, thereby supporting workload prioritisation and risk-based review (18, 36). AI has also been applied to support plan review and error detection using multi-institutional data, suggesting potential value for standardising checks across heterogeneous clinical environments (20).

### 3.2 Feasibility determinants for AI implementation in African radiotherapy centres

Evidence from implementation studies and global radiotherapy guidance consistently shows that AI delivers benefit only when embedded within stable infrastructure, trained teams, and clear governance frameworks (37).

#### 3.2.1 Digital and IT infrastructure readiness

AI-enabled radiotherapy depends on a reliable power supply, good network connectivity, image management systems (PACS), and superior computing capacity. However, many African radiotherapy centres operate with intermittent electricity, limited bandwidth, fragmented PACS deployment, and ageing IT hardware, constraining real-time data transfer and system integration (38, 39).

Two deployment pathways are typically considered: local (on-premises) computing and cloud-based solutions. Local deployment offers greater data control but requires upfront investment in servers, maintenance, and skilled IT support, which may be challenging for low-resource centres. Cloud-based platforms can reduce local hardware demands and facilitate remote planning or QA, but are highly dependent on stable internet connectivity and robust data governance frameworks. Minimum digital requirements for safe AI integration include uninterrupted power supply, secure PACS connectivity, routine data backup, and basic cybersecurity safeguards. Several authors advocate a stepwise preparedness model, beginning with digital stabilisation (power, PACS, networking), followed by semi-automated workflows (e.g. auto-contouring with human review), and progressing to more advanced applications such as adaptive radiotherapy only after institutional maturity is achieved (40, 41).

#### 3.2.2 Digital competence and training gaps

Successful AI adoption requires both clinical competence and digital literacy. While African radiotherapy professionals possess strong clinical skills, formal training in AI concepts, data interpretation, and system validation remains limited (42). This gap can reduce confidence in AI outputs and increase the risk of inappropriate use.

Current evidence suggests that targeted, modular upskilling, rather than full retraining, is the most realistic approach. Short-term training (weeks to months) can focus on AI awareness, limitations, and safe interpretation, while medium-term programs (1–3 years) can integrate AI into residency, physics, and radiography curricula (43–45). International initiatives led by the IAEA, private partnership and professional bodies demonstrate that blended learning models combining online education, fellowships, and mentorship are feasible in African settings (15).

#### 3.2.3 Workforce capacity and sustainability

Radiotherapy workforce shortages remain a fundamental constraint across Africa, with severe deficits in radiation oncologists, medical physicists, and radiotherapy technologists (46). AI is often proposed as a solution to these shortages; however, evidence consistently indicates that AI functions best as a capacity multiplier rather than a replacement for trained professionals (16).Automation of repetitive tasks, such as contouring, plan checks, and scheduling, can reduce workload pressure and improve consistency, potentially supporting staff retention and mitigating burnout (47–49). Task-shifting models, where AI-supported workflows enable supervised practice by less specialised staff, have also been proposed, but require clear protocols, accountability, and quality assurance to ensure safety (50). Long-term sustainability depends on retention strategies, including career development, competitive remuneration, access to continuing education, and supportive working environments. Without parallel investment in human resources, AI deployment risks amplifying existing inequities rather than strengthening radiotherapy capacity (49).

#### 3.2.4 Data governance, regulation, and model transferability

Safe and effective AI-assisted radiotherapy (AI-RT) in Africa depends on more than technical performance; it requires trustworthy data governance, fit-for-purpose regulation, and evidence that models generalise across centres. Global AI governance and clinical AI reporting guidance consistently emphasise privacy protection, transparency, accountable oversight, and robust validation before clinical deployment (18, 40). AI-RT systems rely on large volumes of imaging and treatment data (CT/MRI/CBCT, contours, plans, logfiles), which are sensitive health data. In African contexts, regulatory maturity varies substantially across countries, creating uneven protections and responsibilities for data handling (37, 51). At the continental level, the African Union Convention on Cyber Security and Personal Data Protection (Malabo Convention) provides a framework for data protection and harmonisation, supporting lawful processing, confidentiality safeguards, and cross-border cooperation (52). In addition, WHO’s guidance on AI for health highlights ethical obligations around informed consent (where applicable), transparency of data use, and protection against misuse, particularly in settings with structural vulnerabilities (53).

### 3.3 African-specific risks and mitigation strategies

The deployment of AI-assisted radiotherapy (AI-RT) in Africa entails context-specific risks that differ in scale and nature from those encountered in high-income settings. These risks arise from structural constraints in infrastructure, workforce capacity, data ecosystems, and governance frameworks. Addressing them proactively through explicit mitigation strategies (**Table 1**) is essential to ensure that AI strengthens, rather than undermines, radiotherapy quality and equity in African settings (19, 37).

**Table 1:** Summary AI mitigation strategies.

Risk	Primary cause	Potential impact	Mitigation strategy
Pelvic and Abdominal contouring errors	Organ motion, anatomical variability	Dosimetric inaccuracies, target miss, OAR overdose	Hybrid AI-human workflows with mandatory clinician review
Weak IT systems	Unreliable power, limited networking	Workflow interruptions, system downtime	Phased digital readiness and redundancy planning
Skill gaps	Limited AI-specific training	Unsafe or inappropriate AI use	Modular, role-specific AI education
Data bias	Predominantly non-African training datasets	Reduced model accuracy and equity	Regional model training and validation

#### 3.3.1 How mitigation can be realistically implemented

Mitigation strategies must align with current system capacity rather than idealised end-states. Hybrid AI-human workflows, where AI assists but does not replace expert review, are the most immediately feasible safeguard and are widely recommended in radiotherapy AI implementation literature (16, 47). Infrastructure risks can be addressed through phased digital readiness models, prioritising stable power supply, basic PACS functionality, and secure networking before deploying more advanced AI applications such as adaptive radiotherapy (49, 54). Parallel investments in modular AI education, through short courses and blended learning, can effectively address competence gaps without extensive retraining. Establishing regional validation and training hubs provides a practical solution for data bias and generalisability issues. These hubs facilitate dataset curation, external validation of AI models, and controlled retraining with representative data, enhancing safety and equity. Additionally, strengthening cancer registries and standardising data collection will improve the relevance of AI training and clinical outcome monitoring (19, 35, 37, 44).

#### 3.3.2 Lessons from Africa

Comparative analysis reveals that AI adoption in radiotherapy is influenced more by systems maturity, governance, and workforce capacity than by algorithmic sophistication. Insights from Africa, Europe/North America, and Asia indicate that successful AI integration occurs in digitally mature environments with strong PACS integration and established QA and regulatory processes (49, 55).

#### 3.3.3 Africa: pilot-based innovation within fragile systems

Across Africa, AI-assisted radiotherapy initiatives remain largely pilot-driven, often introduced through partnerships, research collaborations, or vendor-supported deployments. These pilots demonstrate feasibility and efficiency gains, particularly in auto-contouring, planning support, and workflow optimisation, but are frequently constrained by infrastructure fragility, workforce shortages, and limited regulatory clarity (19, 49). Some key examples include: the Johannesburg remote planning protocol which is an ongoing tele-RT project that uses digital workflows enhanced with artificial intelligence for auto-contouring and auto-planning to extend expert capacity to regional centres (56).

The African AI4Radiotherapy (AI4RT) Initiative, a collaborative effort among medical physicists, oncologists, and AI researchers, aims to implement AI-assisted radiotherapy solutions in low-resource environments. This initiative focuses on AI-driven automation in tumour segmentation, treatment planning, and QA to improve accessibility and efficiency in cancer treatment across the continent (19).

**Figure 1:**
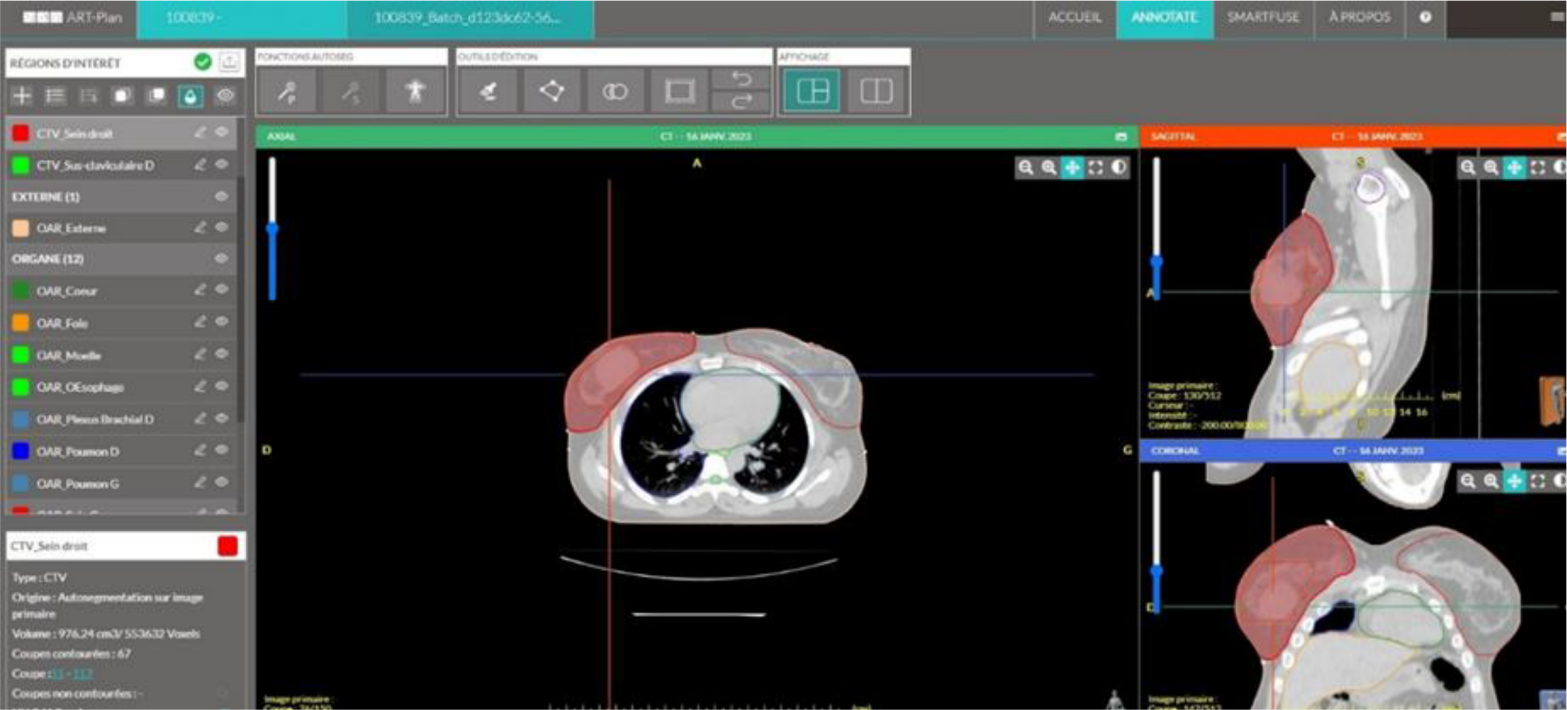
Axial, sagittal, and coronal views of an AI-automated breast target volumes and its associated organs at risks contoured using ART-PLAN software. Future directions for AI-assisted radiotherapy (AI-RT) in Africa include: the integration of imaging and genomic data for precision treatment, the use of AI-enabled tele-radiotherapy to extend access to specialist expertise, the establishment of regional AI hubs for tool validation and training, and the promotion of sustainable innovation through open-source solutions developed in collaboration with African institutions.

Another promising case study is the use of AI-powered radiotherapy planning tools in Egypt’s National Cancer Institute (NCI). Egypt has been at the forefront of AI-driven cancer research, with the NCI integrating AI-based automation in contouring tumour volumes and optimizing radiation dose delivery (57). These AI tools have significantly reduced planning times, enhanced consistency in tumour segmentation, and improved overall workflow efficiency (57, 58). Given the high burden of cervical cancer in Sub-Saharan Africa, AI-driven brachytherapy planning has shown great potential in reducing treatment delays and ensuring equitable access to high-quality radiotherapy and this is being considered to be implemented at the Kenyatta National Hospital in Kenya (59, 60). Through local-foreign private partnerships in Senegal, Togo and Malawi, fully AI-powered web-based solutions for automatic contouring and image fusion (ART-Plan, TheraPanacea as depicted in **Figure 1**) are utilised in three radiotherapy facilities. This intelligent software enables high-precise radiotherapy by optimizing key steps along the treatment workflow (61).

## 4 Conclusion

Artificial intelligence has the potential to enhance radiotherapy for pelvic and abdominal cancers in Africa by improving workflow efficiency and addressing specialist shortages. However, successful implementation depends on factors such as infrastructure, workforce capacity, and data governance. A cautious, feasibility-first approach should prioritise clinical applications like assisted contouring and remote planning while ensuring human oversight. Complex applications should only be pursued when foundational frameworks are established. Sustainable integration will require strategic investment in infrastructure, workforce development, and research. When combined with effective governance, AI can amplify human expertise and improve access to quality radiotherapy in Africa.
